# Tailoring the structural and textural properties of MCM-41 via myristyl trimethyl ammonium bromide templating for enhanced tetracycline adsorption

**DOI:** 10.1038/s41598-026-67402-3

**Published:** 2026-09-02

**Authors:** Mohammed A. Wahba, Rabab K. Khaled, M. F. Zawrah, Magdah Dawy

**Affiliations:** 1 Department of Inorganic Chemistry, National Research Centre, 33 El Bohouth St. (Former Eltahrir St.), Dokki, Giza 12622 Egypt; 2 Department of Physical Chemistry, National Research Centre, 33 El Bohouth St. (Former Eltahrir St.), Dokki, Giza Egypt; 3 Department of Refractories, Ceramics and Building Materials-Center of Excellence for Advanced Sciences, National Research Centre, 33 El Bohouth St. (Former Eltahrir St.), Dokki, Giza Egypt

**Keywords:** Mesoporous silica, MCM-41, Myristyl surfactant, Tetracycline removal, Adsorption, Chemistry, Environmental sciences, Materials science

## Abstract

The escalation of pharmaceutical water pollution, particularly from antibiotics, poses a critical environmental crisis. Consequently, developing highly efficient adsorbents has become imperative to tackle this growing threat. Here, highly ordered pure MCM-41 was synthesized using different molar concentration ratio (0.06, 0.12, 0.25 and 0.415 M) of myristyl trimethyl ammonium bromide surfactant to the silica source. The structural, morphological and texture characteristics were investigated by X-ray diffraction (XRD), N_2_ adsorption–desorption, scanning electron microscopy (SEM), transmission electron microscopy (TEM) and Fourier transform infrared (FTIR). It was found that the myristyl concentration has a profound impact on the mesostructural arrangement of the particles. Consequently, it was found that the highest quality and highly ordered pure MCM-41 structure was achieved using myristyl surfactant at concentrations of 0.25 M and 0.12 M, with the 0.25 M concentration producing a higher degree of ordered pure MCM-41 structure than the 0.12 M concentration. The potential of the prepared MCM-41 samples for adsorption of tetracycline (TC) drug was investigated using varying parameters including: adsorbent dosage, initial drug concentration, adsorption isotherms, and contact time. The MM0.25 sample showed remarkable TC-adsorption removal, with maximum adsorption capacity (q_max_) of 5.4 mg g^−1^ at pH 5.4 and room temperature. The adsorption equilibrium data fitted well with Langmuir adsorption model, while the adsorption kinetic followed the pseudo-second-order model (R^2^ = 0.99999). The calculated free adsorption energy obtained from D–R isotherm was found to be 1507.6 kJ mol^−1^ referring to that the adsorption of TC on MM0.25 adsorbent is chemical. These results revealed that MM0.25 sample could be used as effective adsorbent for the removal of TC drug from aqueous solutions with good recovery and recyclability.

**Supplementary Information:**

The online version contains supplementary material available at https://doi.org/10.1038/s41598-026-67402-3.

## Introduction

Freshwater, our planet's most critical resource, is facing an escalating threat from diverse pollutants. According to the World Health Organization (WHO)/UNICEF Joint Monitoring Programme (JMP), approximately 25% (1 in 4 people, or ~ 2.1 to 2.2 billion people) of the global population lacks access to safely managed drinking water services which highlights the critical need for advanced water treatment technologies^1^. Among these contaminants, tetracycline is considered the world’s second most widely used antibiotic and its residues present a severe challenge. Due to widespread overuse in human medicine and livestock and its incomplete metabolic breakdown, TC residues frequently contaminate surface water, rainwater, soil, and drinking water. TC persists in the environment as it is highly chemically stable and resistant to biodegradation. This poses significant risks to both human health and ecological systems^2,3^. Consequently, developing effective methods to remove TC-residuals is an urgent priority. Currently, a variety of chemical, biological, and physical techniques including coagulation, flocculation, membrane separation, and adsorption^4^ have been employed in antibiotics eliminations process. Among these techniques, physical adsorption stands out as one of the most effective strategies for diverse pollutants removal^5–8^.

Interest in mesoporous materials has grown significantly since 1992, when the Mobil Oil Corporation reported the first M41S family of mesoporous silica^9^. Among these materials, the MCM-41 molecular sieve has been extensively studied due to its well-defined arrangement. These materials excel with unidirectional uniform and controllable diameters hexagonal mesopores^10^. The large specific surface area (SSA), narrow adjustable pore size, and high adsorption capacity of this highly ordered material magnifies its widely utilizations in catalysis, adsorption^11^, and catalyst support applications^12,13^.

In general, MCM-41 synthesizing molecular sieve has driven extensive research focusing on simultaneously controlling of its morphology and texture. Crucial reaction parameters including the solvent choice, reaction temperature, conditions of aging and drying, stirring rates, pH, silica precursor, type of surfactant, and the surfactant to silica ratio play a critical role in tailoring the size and internal structure shape of MCM-41 for specific applications^14–16^. Specifically, MCM-41 materials are synthesized via the hydrothermal transformation of a basic silicate solution in the presence of quaternary ammonium surfactants featuring varying alkyl chain lengths^12^. A wide range of surfactants including cationic^17^, anionic^18^, and nonionic^19^ surfactants, as well as amphiphilic block copolymers^20^ have been successfully utilized to synthesize mesostructured materials^21^. The surfactant concentration exerts a profound impact on the mesostructural arrangement of the resulting particles. While the lower concentration of surfactant fails to initiate micelle formation leading to template deficient nanoparticles, conversely, the too high concentration can disrupt the ordering, resulting in a disordered framework^22^.

Therefore, the aim of this work was to prepare highly ordered mesoporous MCM-41 with myristyl trimethyl ammonium bromide surfactant and utilizing the prepared mesoporous MCM-41 for the removal of TC drug present in aqueous solutions. Firstly, highly ordered mesoporous MCM-41 was synthesized via an autoclave assisted hydrothermal method. The effect of different molar concentration ratio (0.06, 0.12, 0.25 and 0.415 M) of myristyl surfactant to the silica source was investigated. The influence of the surfactant molar concentration ratio on the structural, morphological and textural properties of the MCM-41 framework has been investigated. The potential of the prepared highly ordered pure MCM-41 using myristyl surfactant (MM0.12 and MM0.25 samples) for adsorption of TC drug was investigated. The influence of initial TC drug concentration, adsorbent dosage and contact time was investigated. Langmuir and Freundlich models were applied to study the equilibrium isotherms. The suitability of the pseudo-second-order kinetic model for the description of the kinetic data was investigated, from which the adsorption mechanism was examined.

## Experimental

### Materials and reagents

Tetra decyl trimethyl ammonium bromide (Myristyl trimethyl ammonium bromide, CH_3_(CH_2_)_13_N(CH_3_)_3_Br), 99%, MW = 336.41, Sigma-Aldrich Chemie GmbH, Germany. Tetra ethyl orthosilicate (TEOS, C_8_H_20_O_4_Si), MW = 208.33 g/mol, was purchased from Merck, Germany. Sodium hydroxide pellets, MW = 40 g/mol, was purchased from SIGMA-ALDRICH. TC drug (Commercial tetracid drug, C_22_H_24_N_2_O_8_), CID (Chemical Industries Development), Egypt. All used chemicals were of analytical grade. All chemicals and prepared solutions were handled in accordance with appropriate safety protocols and standard laboratory safety procedures, following the supplier Safety Data Sheets (SDS) (including the use of personal protective equipment, fume hoods, and compliant waste disposal procedures).

### Preparation of ordered pure mesoporous MCM-41 by autoclave assisted hydrothermal method

Four different samples of pure mesoporous MCM-41 were prepared using an autoclave assisted hydrothermal method while the myristyl surfactant was used as the template at different molar concentration ratio (0.06, 0.12, 0.25 and 0.415 M) of the surfactant to the silica source and TEOS was used as the silica source.

0.5 gm NaOH was dissolved in 89.7 mL of distilled water. Then, the desired weight (1.68, 3.35, 6.98 and 11.59 gm) of myristyl surfactant according to the molar concentration ratio (0.06, 0.12, 0.25 and 0.415 M) was added then the mixture was kept under stirring for 1 h. at 60 °C and 350 rpm till complete dissolving. A volume of 17.3 mL of TEOS was added drop by drop, the mixture was kept under stirring at pH 10 for 1 h. at room temperature and 350 rpm. Finally, the solution was transferred to the autoclave at 110 °C for 48 h. The sample was washed with dist. H_2_O and ethanol several times, then dried at 70 °C. The sample was calcined at 550 °C for 6 h. The obtained materials were coded as MM0.06, MM0.12, MM0.25 and MM0.415, respectively according to the molar concentration ratio (0.06, 0.12, 0.25 and 0.415 M)^11^.

### Characterization

The characterization of the materials was performed using X-ray diffraction (XRD) on (XRD, PANalytical X-ray diffraction equipment model X′Pert PRO). N_2_ adsorption–desorption isotherms were measured at − 196 °C using a NOVA 3200 instrument (USA) to calculate specific surface area (S_BET_), pore size distribution (using DFT method), and pore volume. Initially, the samples were degassed under vacuum (10^−4^ Torr) at 300 °C overnight. The particles morphology and size were analyzed using a scanning electron microscope (SEM, model Quanta 250 FEG) and transmission electron microscope (TEM, JEOL JEM-2100). The functional groups present in the samples were identified through FTIR transmittance spectra using FTIR-ATR Brucker Vertex 80 V from Bruker Optik GmbH, Germany in the range of 4000–400 cm^-1^ with resolution 4 cm^-1^.

### TC drug removal

Firstly, to establish the standard calibration curve, a series of TC standard solutions (5, 10, 20, 30, 40 and 50 mg L^-1^) were analyzed using a Cary 100 UV–Vis spectrophotometer at λ_max_ = 358 nm and then, the calibration curve was presented in Figure S1. Also, 500 mL of 100 mg L^-1^ TC stock solution was prepared. The highly ordered pure MCM-41 prepared using myristyl surfactant (MM0.12 and MM0.25 samples) were evaluated for the adsorption experiments of TC drug. The effect of dose was studied using varied dosage of the MM0.12 and MM0.25 adsorbents (1–5 g L^-1^) in 50 mL of 10 ppm of TC. The adsorption experiments of TC drug was done by performing batches containing 250 mg of MM0.25 sample in 50 mL of initial concentrations (5–50 mg L^-1^) of the drug, and these batches were shaken at 250 rpm in a bath shaker at room temperature. The concentration of remaining TC drug was determined employing the maximum absorbance at 358 nm, using a Cary 100 UV–Vis spectrophotometer. The removal drug efficiency percentage (R%) and the adsorption capacity q_e_ (mg g^-1^) were calculated^23^.

For kinetic measurements and adsorption isotherms, where the adsorption equilibrium could be reached, the solutions were collected at different intervals in a range of 2–120 min by adsorption of 10 mg L^-1^ TC solutions on 250 mg of MM0.25 sample. The interactive behavior between TC molecules and MM0.25 sample could be revealed by an equilibrium adsorption isotherm which is an important part of the design of adsorption systems so, two common isotherm models were used; Langmuir and Freundlich models. The Kinetic measurements including the pseudo-first-order, pseudo-second-order and intra-particle diffusion (IPD) models were used to test the experimental data. All experiments were conducted in triplicate, with consistent results across three independent trials.

## Results and discussion

### XRD analysis

**Fig. 1:**
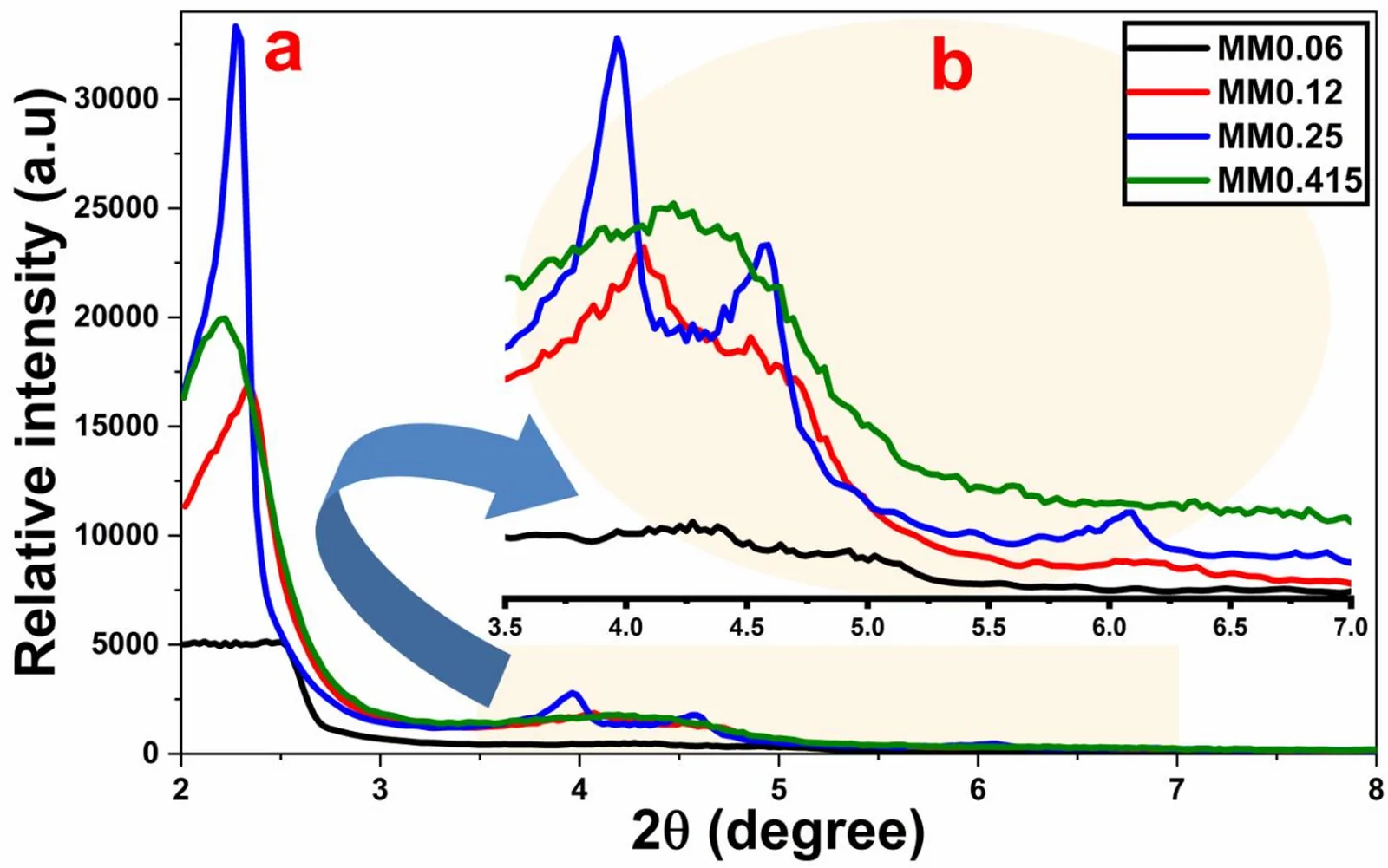
(**a**) XRD plot for MM0.06, MM0.12, MM0.25 and MM0.415 samples, (**b**) magnified pattern shows the less intense XRD peaks of the samples.

XRD patterns for the pure MCM-41 samples with different concentrations (0.06, 0.12, 0.25 and 0.415 M) of myristyl surfactant are presented in Fig. 1. A well-formed MCM-41 typically exhibits at least three obvious peaks in the range 2 and 8° 2θ. Here, the XRD patterns exhibit three to five Bragg peaks depending on the surfactant concentrations. These peaks correspond to the (100), (110), (200), (210) and (300) reticular planes of a hexagonal packing of the pores, and are typical of MCM-41 type materials. The MM0.25 sample (Fig. 1) showed a set of five prominent peaks referring to a well-formed MCM-41 structure. The first three peaks 2.3, 3.9 and 4.6 are related to (100), (110), and (200) Miller indices of a hexagonal unit cell and referring to well-ordered hexagonal mesopores^24^. Two extra peaks, observed at 6.1 and 6.,9 related to 210 and 300 Miller indices, respectively, is occasionally detected in exceptionally well-ordered MCM-41, indicating successful synthesizing of highly ordered MCM-41^11^. Some variances could be noted comparing XRD spectra of MM0.25 with the other concentrations; as a general trend, there is a broadening of the diffraction peaks accompanied by shifts to higher 2theta degrees in case of MM0.06 and MM0.12 samples and lower 2theta degrees in case of MM0.415 sample. Deviations outside of this range resulted in a decrease of the pore mesostructure ordering due to the disturbing of the assembly on MCM-41 structure during the synthesis^25^. Also, the most prominent peak labeled at 100 for MM0.06, MM0.12 and MM0.415 samples was not as sharp as that of MM0.25 sample. The less intense diffraction peaks of MM0.06, MM0.12 and MM0.415 samples comparing to the MM0.25 sample would be related to a wider distribution of pore diameter and less ordering in the arrangement of mesopores.

For MM0.06 sample, the intensity of the three peaks (100), (110) and (200) was highly diminished. For MM0.12 sample, the most prominent peak labeled as 100 was shifted towards higher 2theta degrees (2θ = 2.3). Similar conduct was observed for the three peaks (110), (200) and (210) and the other peak (300) was disappeared. However, the MM0.12 sample still demonstrates the main diffraction peaks, although the intensity of the (110), (200) and (210) peaks are reduced; they are still detected and the (110) and (200) peaks are overlapped (Fig. 1b). For MM0.415 sample, the peak (100) became broader and the two peaks (110) and (200) were overlapped at 2θ = 4.2. This behavior could be related to that higher surfactant concentrations inhibit unit cell growth and reduce silica polymerization, ultimately disrupting the well-ordered hexagonal structure of the MCM-41 silica^25^. Coleman and Attard investigated the effects of surfactant concentration on the regularity and morphology of mesoporous silica. They observed that very low surfactant concentrations (e.g., sample MM0.06) produced a disordered structure. However, increasing the concentration (as in samples MM0.12 and MM0.25) rapidly enhanced structural order, yielding a well-defined hexagonal mesopore structure. Beyond this point, further increases in surfactant concentration (e.g., MM0.415 sample) progressively disrupted the hexagonal order. They attributed this behavior to the formation of lyotropic liquid crystalline phases in the surfactant–water binary system; the structural order of the mesoporous silica peaked at the exact surfactant ratio where the hexagonal phase emerges in the binary system^26^. Consequently, the XRD pattern showed that the highest quality and highly ordered pure MCM-41 was formed at 0.25 M and 0.12 M concentrations of the myristyl surfactant however the 0.25 M concentration produced higher degree of ordered pure MCM-41 structure than the 0.12 M concentration.

XRD results were applied to calculate the distance between two neighboring pores “the cell parameter a_0_” based on the 2θ value of the (100) peak and Bragg’s Law: ${\mathrm{a}}_{0}= 2{\mathrm{d}}_{100}/\sqrt{3}$. Also, the primary mesopore diameter (W_d_) was calculated using the values of cell parameter according to the equation ${\mathrm{W}}_{\mathrm{d}}=\mathrm{c}\mathrm{d}\sqrt{(\uprho {\mathrm{V}}_{\mathrm{p}}/(1+}\uprho {\mathrm{V}}_{\mathrm{p}})$ (the constant ‘c’ = 1.213, d represents the d-spacing of the (100) peak, ρ refers to the density of the pore wall, V_p_ is the volume of a primary mesopore)^27,28^. The values of the d_100_-spacing, a_0_, W_d_ and W_t_ are determined and summarized in Table 1. It was found that the values of d_100_-spacing, a_0_, W_d_ and W_t_ increased by increasing the myristyl concentration.

**Table 1:** Structural and textural properties of the samples.

Samples	XRD				N_2_ adsorption–desorption isotherms			TEM	
	d_100_ (nm)	a_0_ (nm)	W_d_ (nm)(XRD)	W_t_ (nm)	S_BET_ (m^2^.g^−1^)	V_p_ (cm^3^ g^−1^)	D_p_ (nm)	a_0_ (nm)	W_d_ (nm) (TEM)
MM0.06	3.63	4.19	3.50	0.69	846	0.64	2.8	3.23	2.54
MM0.12	3.79	4.37	3.65	0.72	808	0.69	3.9	4.20	3.48
MM0.25	3.88	4.48	3.74	0.74	863	0.72	3.5	4.50	3.76
MM0.415	3.97	4.59	3.83	0.76	973	0.76	3.9	3.19	2.43

(a_0_) average lattice parameter: a_0_ = 2d_100_ /√3; V_p_: pore volume; D_p_: most probable pore size, calculated by DFT model; W_t_: Wall thickness = a_0_ − D_p_.

### N_2_ adsorption–desorption isotherms

**Fig. 2:**
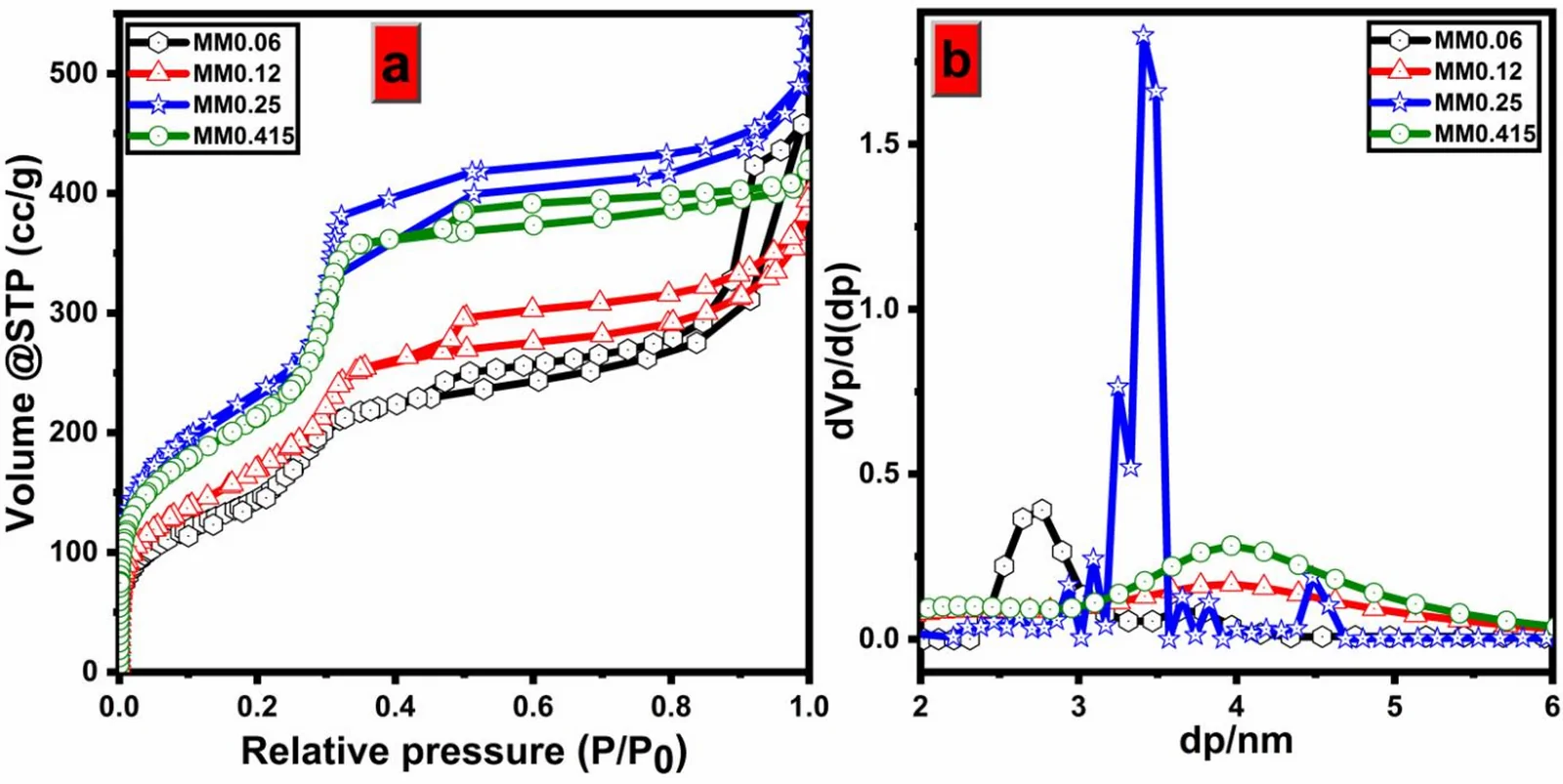
(**a**) N_2_ adsorption–desorption isotherms and (**b**) pore size distribution of MM0.06, MM0.12, MM0.25 and MM0.415 samples.

The textural parameters of prepared samples were studied using the nitrogen adsorption method. Figure 2a depicts the full N_2_ adsorption–desorption isotherms for all samples over varying P/P_o_ pressures from 0 to 1.00. All the synthesized materials demonstrated type IV isotherms^29^. In the low pressure (P/P_o_ < 0.1), all samples displayed a sharp initial uptake of nitrogen indicating the presence of micropores or a high primary adsorption on the surface^30^. In the intermediate pressure (P/P_o_ = 0.3 to 0.4), there is a noticeable "step" or inflection point, particularly prominent in the MM0.25 sample, representing capillary condensation within the mesopores. Notably, a characteristic H4 hysteresis loop was observed in the range of 0.40–0.99 for all samples attributed to capillary condensation within the mesopores. At the higher pressures, the curve rises sharply and the lack of a plateau as the relative pressure approaches 1.0 suggests that the adsorption saturation was not reached. The specific surface area of the MM0.06, MM0.12, MM0.25 and MM0.415 samples was found to be 846, 808, 863 and 973 m^2^ g^-1^, respectively (Table 1) and this indicated that the surface area increased with increasing the concentration of myristyl surfactant from 0.12 to 0.415 M. This behavior is attributed to the increased adsorption of positive micelles onto the negatively charged silica cores at higher surfactant concentrations. Following TEOS hydrolysis and polycondensation, these micelles assemble with the inorganic species to form porous layers, leading to a substantial increase in both specific surface area and pore volume^31^. Furthermore, the total pore volume tended to increase with higher myristyl concentrations, a trend that aligns with previous findings^32,33^.

Plots of DFT pore size distribution for MM samples are presented in (Fig. 2b). All samples exhibited a variation in pore size distribution; however, a notably narrow mesopore size distribution was detected in the MM0.25 sample (Fig. 2b). The lower uniformity in pore size across the other samples suggests a higher degree of structural disorder. This aligns with the XRD data and may be attributed to either an asymmetrical pore arrangement, a higher concentration of defects within the pore walls, or both. Textural features of the synthesized materials were attained from the adsorption–desorption isotherms results and are depicted in Table 1. Generally, XRD results are in good agreement with the results of N_2_ adsorption and confirms the well-ordered structure for the samples especially MM0.25 sample.

### SEM

**Fig. 3:**
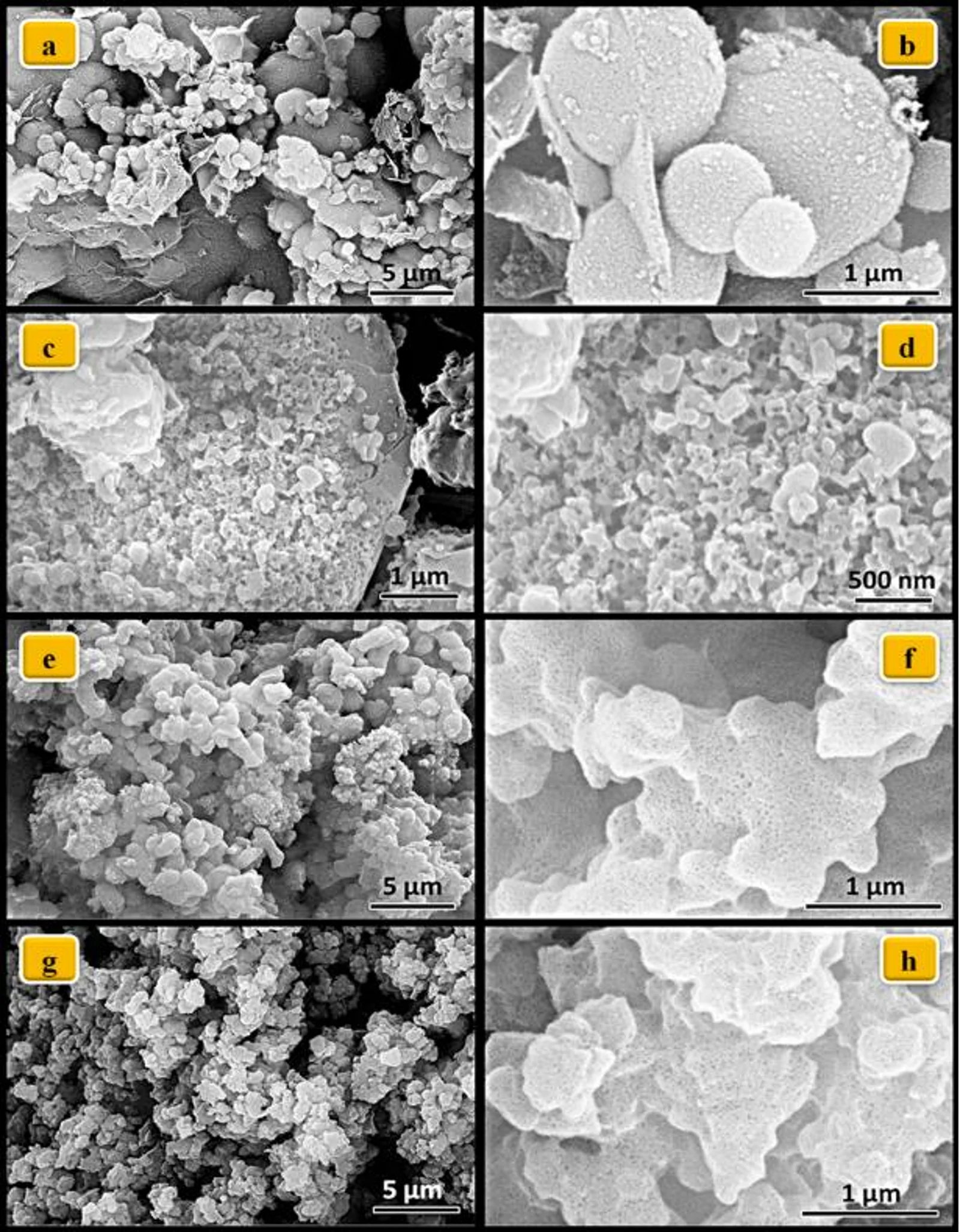
SEM images of MM0.06 sample (**a**,**b**); MM0.12 sample (**c**,**d**); MM0.25 sample (**e**,**f**) and MM0.415 sample (**g**,**h**).

Figure 3 depicts the scanning electron microscope (SEM) images of prepared MCM-41 samples. MM0.06 sample showed a well-defined spherical morphology (Fig. 3a,b) with particle size range from 300 to 500 nm whereas the other samples exhibited an irregular morphology without defined form and smaller particle size (Fig. 3c–h). The average diameter of the particles was estimated to be 72.4 nm for MM0.12 sample. It was found that the smaller size of silica particles is related to the increase in myristyl concentration. Increasing myristyl concentration promotes the formation of micellar structures, thereby enhancing the bonding density between silica and the myristyl surfactant. Conversely, at lower concentrations, unbound myristyl interacts primarily with Si–O-Si siloxane groups, leading to an increase in silica particle size^34^. Zhao et al. described the mechanism of mesoporous material formation, wherein the part of the Rn-N^+^(R)_3_ group will bind to silica throughout its surface. Then, silica will form a long chain of Si–O-Si which will become a particle^35^.

Also, it was observed that when the surfactant concentration increases, the grooves and pores on the surface of the prepared mesoporous silica gradually increase. Consequently, SEM images of MM0.12, MM0.25 and MM0.415 samples (Fig. 3c–h) shows that the surface pores are relatively abundant and orderly, which provides sufficient adsorption sites for wastewater adsorption^36^.

### HRTEM

**Fig. 4:**
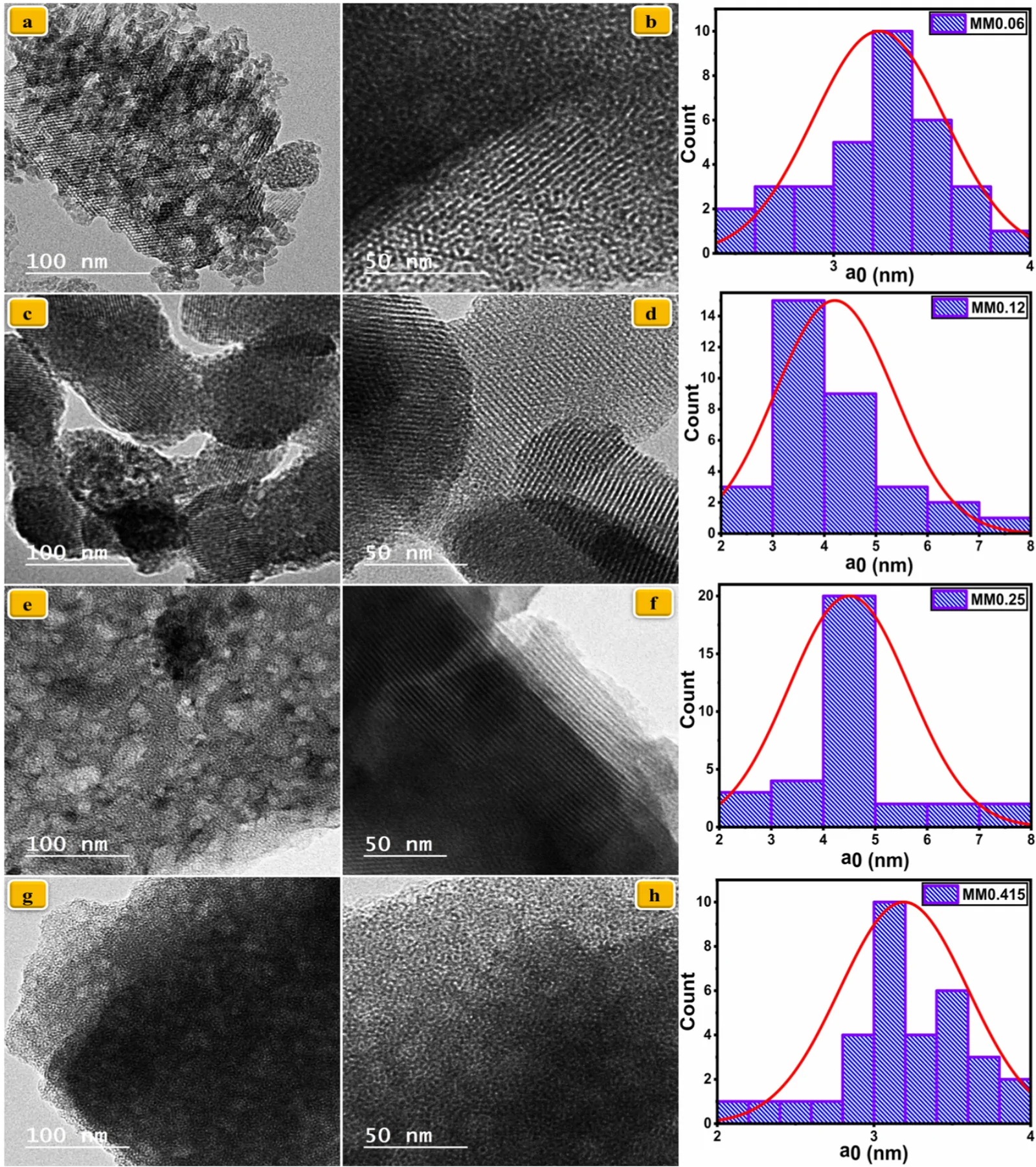
The HRTEM images of MM0.06 sample (**a**) honeycomb-hexagonal array, (**b**) well-ordered pattern; MM0.12 sample (**c**) well-ordered pattern, (**d**) well-ordered pattern; MM0.25 sample (**e**) honeycomb structure, (**f**) well-ordered pattern and MM0.415 sample (**g**) honeycomb structure, (**h**) honeycomb structure with high magnification and histogram represented the average cell parameter ‘a_0_’ of the samples.

The TEM images of MM0.06, MM0.12, MM0.25 and MM0.415 samples are shown in Fig. 4. TEM of the samples plainly showed some key features of MCM-41 and the presence of pores scattered throughout the surface of the silica particles^37^. TEM images of MM0.06 (Fig. 4a) clearly showed a distinctive hexagonal pore structure depicting each pore surrounded by six neighbors. TEM images of MM0.06, MM0.12 and MM0.25 samples (Fig. 4b–f) showed that the silica microspheres displayed a pseudo-periodic lattice of a parallel bundle of pores. Also, a series of parallel lines of alternating dark and light (parallel fringes) representing the repeating pattern of MCM-41 pores were observed referring to a long-range ordered structure of the one-dimensional channels of MCM-41 structure and matched well with the XRD results^11^. On the other hand, the TEM images of MM0.415 sample shown in (Fig. 4g,h) presented a similar pore shape related to the other samples. However, partially irregular pore-ordering and morphological defects were observed which probably related to a partial collapse of the structure of MCM-41. The hexagonal array and regularity of the pore channels were more pronounced in the TEM images of MM0.12 and MM0.25 samples and were in good agreement with both XRD and N_2_ adsorption isotherms. The distance separated between the centers of lighter spots representing the cell parameter ‘a_o_’ was measured, between at least 50 centers, from the TEM images (Fig. 4). Also, the average pore diameter (W_d_) was estimated by substracting the a_o_ value calculated from TEM from the average thickness of the pore wall (W_t_) calculated from XRD^27^. The a_0_ and W_d_ values were summarized in Table 1. It was found that the values of a_0_ and W_d_ were well in line with the average a_0_ and W_d_ values, respectively determined via X-ray diffraction.

### FT-IR

**Fig. 5:**
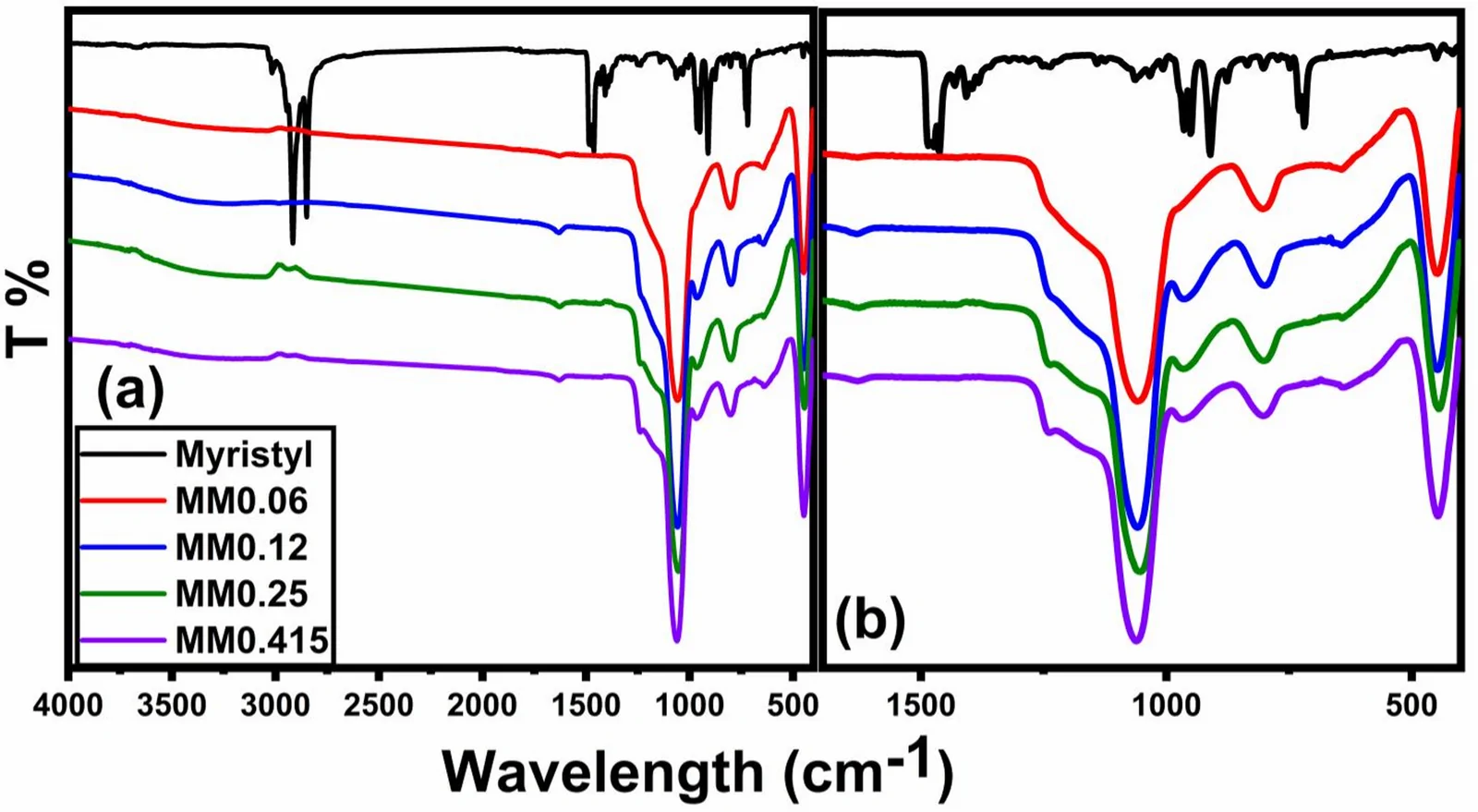
(**a**) FT-IR spectra (400–4000 cm^−1^) and (**b**) magnified FT-IR spectra (400–1700 cm^−1^) of myristyl surfactant, MM0.06, MM0.12, MM0.25 and MM0.415 samples.

The FT-IR of myristyl surfactant and MM samples were depicted in Fig. 5. The myristyl surfactant exhibited various bands at 3010.12 and 2912.45 cm^-1^ (asymmetric stretching vibrations of CH groups), 2850.07 cm^-1^ (symmetric stretching vibrations of CH groups) and 1476.22 and 1461.10 cm^-1^ (the bending modes of δCH(CH_3_) and δCH(CH_2_) groups)^38,39^.

For MM samples, all the bands characteristic to myristyl surfactant disappeared and this confirmed the complete removal of myristyl surfactant. The samples showed bands in the region of 400–4000 cm^-1^ characteristic to the vibration of the MCM-41 structural network and consistent with the literature^30,40,41^. The band at 1062.38 cm^-1^ (for MM0.06, MM0.12 samples) and two bands at 1236.39, 1054.99 cm^-1^ (for MM0.25, MM0.415 samples) were assigned to asymmetric (Si–O–Si)_as_. While the band at 963.89 cm^-1^ was assigned to υ_ass_(SiOH) for all samples but was absent for MM0.06 sample. The band observed at 803.83 cm^-1^ for all samples was related to υ_ss_(Si–O–Si). The well-defined bands at 642.95, 447.61 cm^-1^ (for MM0.06 and MM0.12 samples) and 636.39, 447.61 (for MM0.25 and MM0.415 samples) were characteristic to bending mode of siloxane δ(Si–O–Si). Combining the data obtained from XRD, SEM, HRTEM, and surface area and porosity analyses, it was found that the myristyl concentration has a profound impact on the mesostructural arrangement of the particles. Also, we can conclude that all analyses confirmed the formation of highly ordered pure MCM-41 for MM0.25 and MM0.12 samples but the MM0.25 sample formed highly ordered pure MCM-41 structure more than the MM0.12 sample.

### Adsorption studies

The adsorptive removal ability of MM0.12 and MM0.25 samples for TC drug at different doses was investigated. The MM0.25 sample remarkably enhanced the TC drug removal efficiency at different doses (1, 2 and 5 g L^-1^) in comparison with the MM0.12 sample. The MM0.25 sample showed affinity to remove TC drug from aqueous solution with removal efficiency 36.6, 63.8 and 86.8% at 1, 2 and 5 g L^-1^ doses, respectively. Whereas, the MM0.12 sample showed removal efficiency 33.6 and 50.3% at 1 and 2 g L^-1^ doses, respectively. The higher affinity of the MM0.25 sample for adsorption of the TC in comparison with the MM0.12 sample could be related to the long-range well-ordered structure and higher surface area of MM0.25 sample as indicated in XRD and N_2_ adsorption isotherms. So, the following experiments were carried out using the MM0.25 sample.

#### Effect of adsorbent dosage

**Fig. 6:**
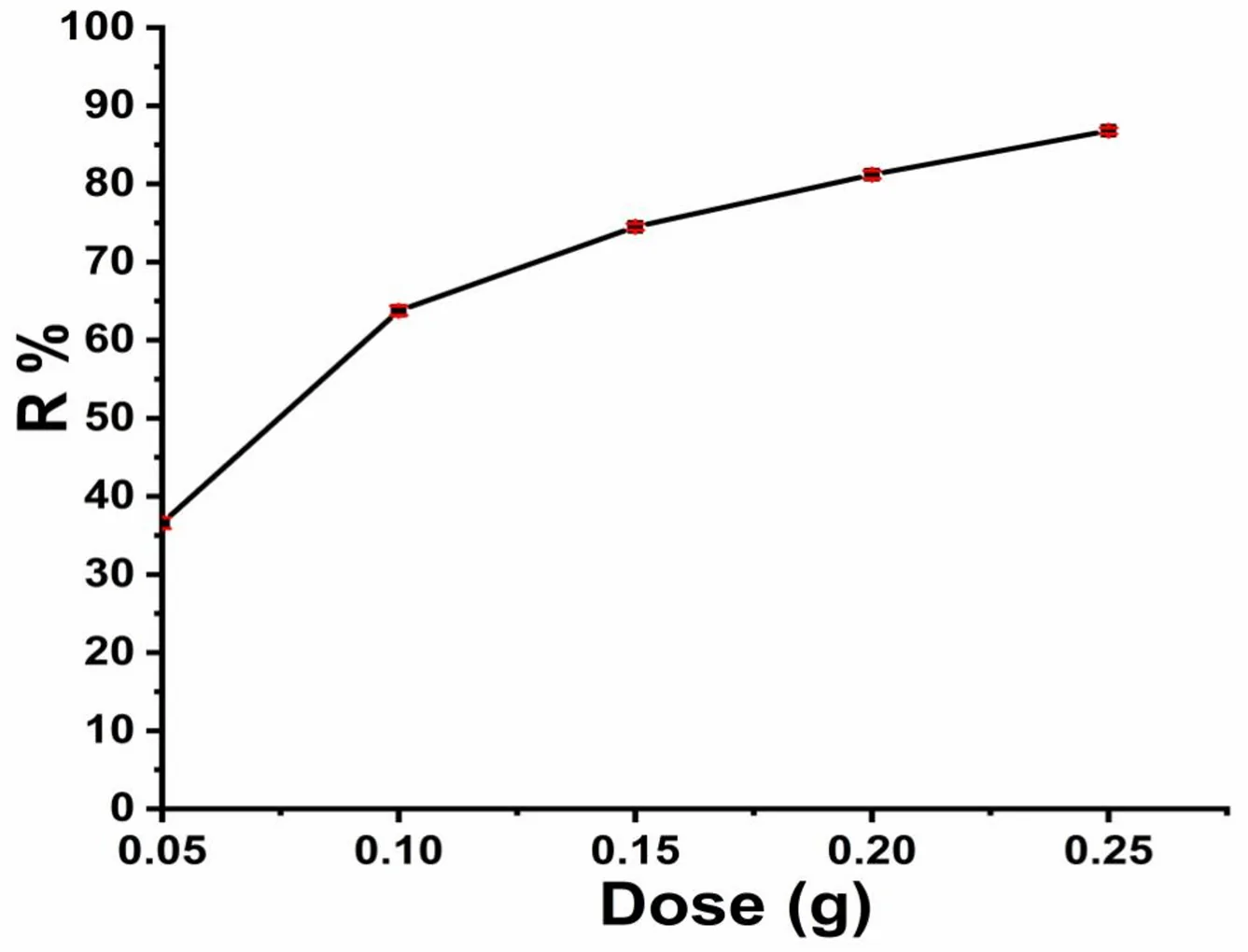
Effect of MM0.25 dosage on TC adsorption (10 g L^−1^ TC concentration, pH 5.4 and room temperature).

The effect of adsorbent MM0.25 dose on the TC removal was shown in Fig. 6. The adsorption removal of TC increased significantly by varying the adsorbent dose from 1 to 5 g L^-1^. It was found that when the dose reached 5 g L^-1^, the adsorption of TC reached the maximum removal efficiency (86.8%) through only 5 min. According to these findings, 5 g L^-1^ of the adsorbent dosage was applied as the optimal dose for the adsorption study.

#### Effect of initial drug concentration

**Fig. 7:**
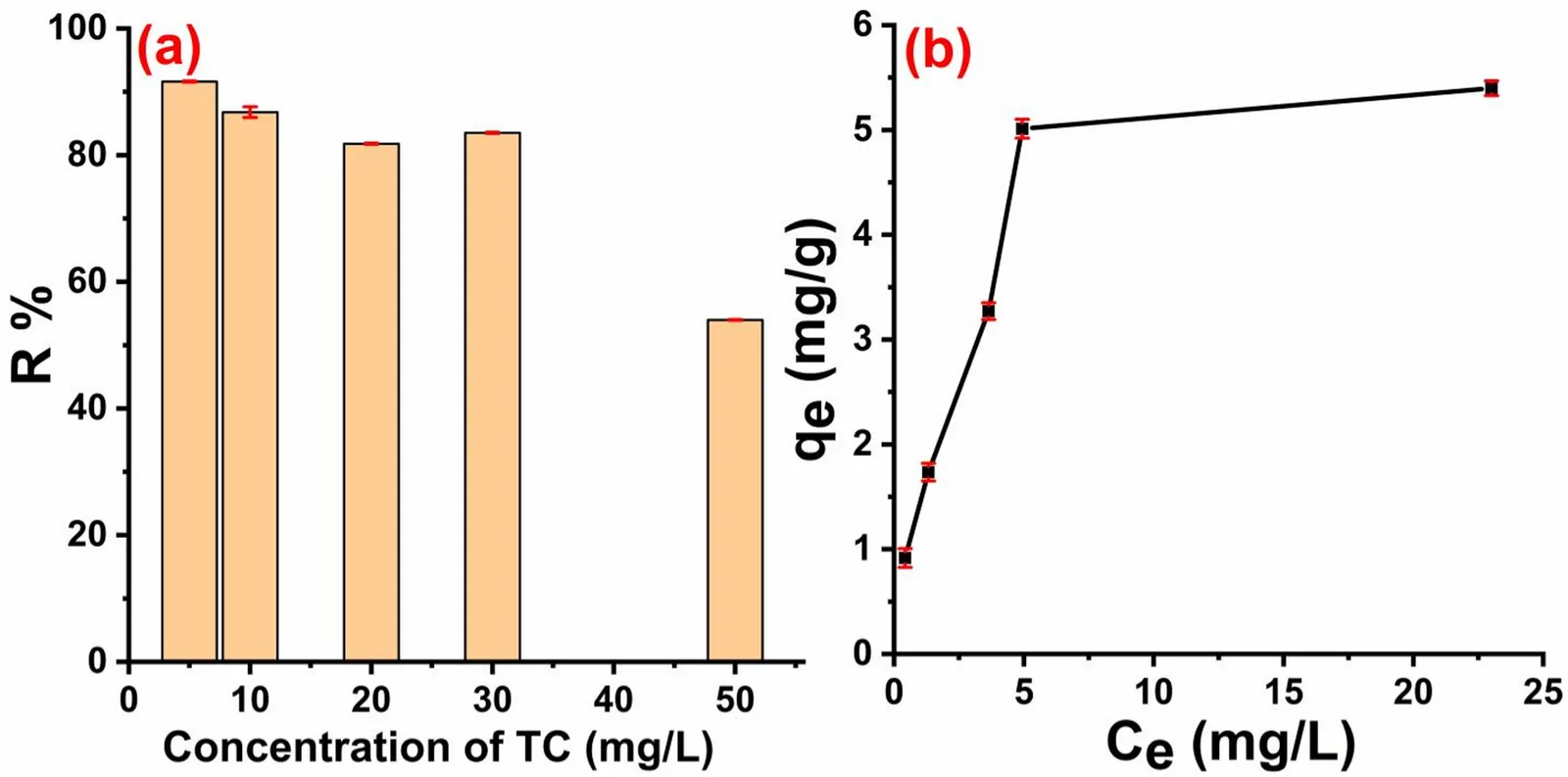
Effect of initial TC concentration on (**a**) the adsorption removal efficiency and (**b**) the adsorption capacity (5 g L^−1^ adsorbent dose, pH 5.4 and room temperature).

The observed adsorption capacity increased with rising initial TC concentrations until equilibrium was reached. To evaluate the effect of initial concentration on adsorption competency, TC concentrations were varied from 5 to 50 mg L^-1^, with the performance of the MM0.25 sample illustrated in Fig. 7. At low initial concentrations, the adsorbent eliminated the vast majority of the drug; for instance, at 5 mg L^-1^, a removal efficiency of 91.6% was achieved within just 5 min. This high efficiency at low concentrations can be attributed to the optimal driving force required to overcome mass transfer resistance as the drug molecules move from the aqueous phase to the MM0.25 surface^42^. Conversely, removal efficiency decreased at higher initial concentrations, which can be explained by the fact that the high initial numbers of drug molecules far exceeded the available active sites on the adsorbent surface^43^.

#### Effect of pH and point of zero charge

**Fig. 8:**
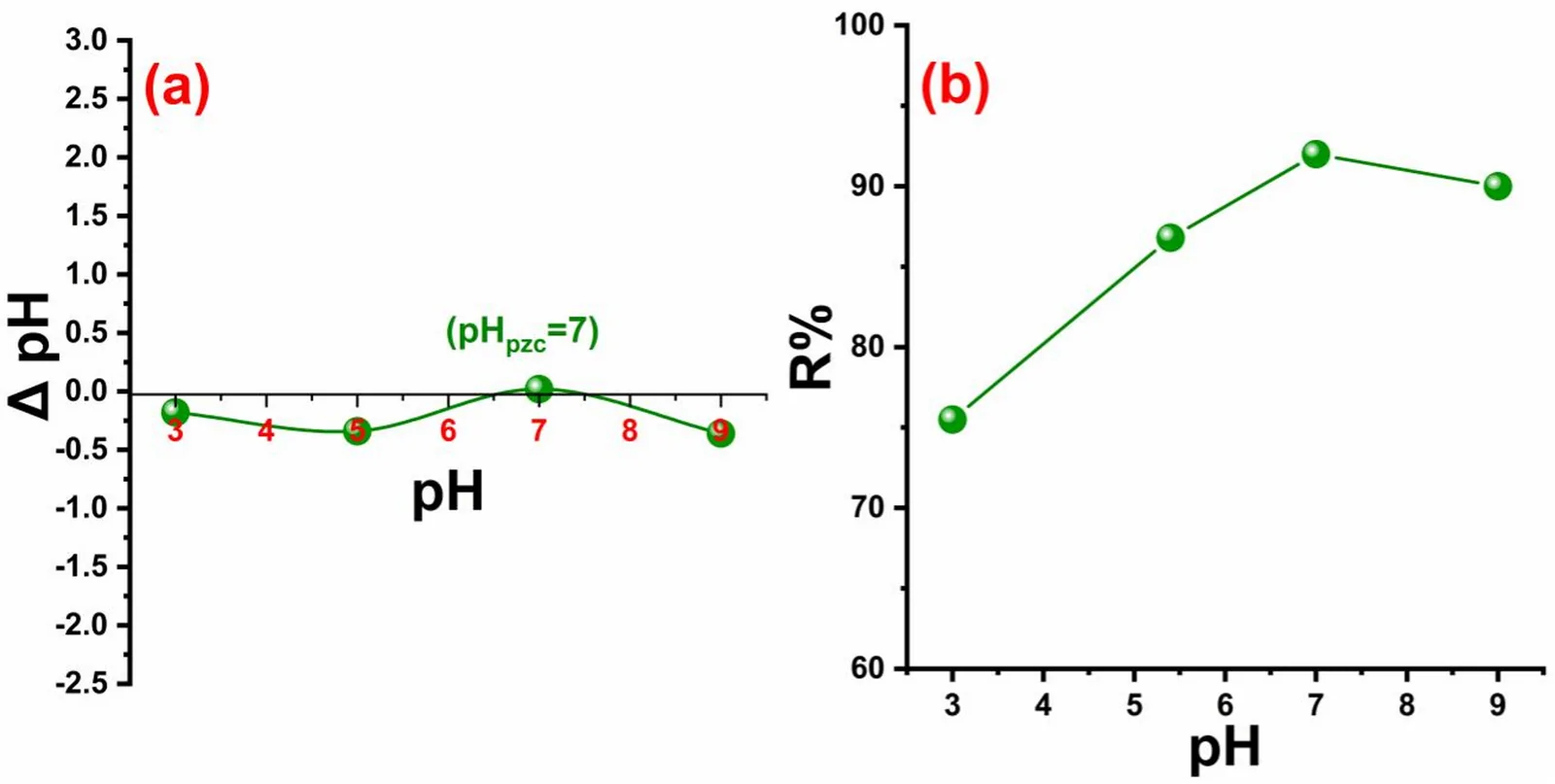
(**a**) Point of zero charge (pH_ZPC_) of MM0.25 and (**b**) the tetracycline removal efficiency of MM0.25 at different pH values.

Figure 8a,b illustrates the point of Zero Charge (pH_zpc_) of MM0.25 sample and effect of pH. It was found that the point of Zero Charge (pH_zpc_) for MM0.25 sample is 7 . A higher PZC value is theoretically linked to improved adsorption performance. The pH of the solution plays a crucial role in influencing the surface charge of the MM0.25 adsorbent and TC molecules, thereby affecting TC adsorption onto the MM0.25 surface and influencing the adsorption efficiency. As depicted in Fig. 8b, the adsorption efficiency of TC using MM0.25 exhibits strong pH dependence. This behavior is attributed to the amphoteric nature of TC, which causes its charge to vary with solution pH. TC removal efficiency increased between pH 3 and 5, peaked at pH 7, and declined from pH 7 to 9. This trend is governed by electrostatic interactions between the pH-sensitive surface charge of MM0.25 and the changing speciation of TC molecules.

TC possesses multiple ionizable functional groups, causing its speciation to shift with pH. Under acidic conditions, TC exists as a cation (H_2_TC^+^), transitions to a zwitterionic form (H_2_TC^0^) between pH 3.3 and 7.7, and becomes predominantly anionic (HTC^−^/TC^2−^) at higher pH values^44^. Within the pH range of 5–7, TC primarily adopts a zwitterionic form, while the MM0.25 surface remains slightly positively charged (pH_zpc_ = 7, Fig. 8a). Although TC is overall neutral in this range, electrostatic interactions occur between its negatively charged tricarbonylamide groups and the positively charged MM0.25 active sites, yielding maximum adsorption. Conversely, at pH values below 5, electrostatic repulsion between the cationic TC species and the positively charged MM0.25 surface hinders adsorption. Above pH 7 (MM0.25’s PZC), the negatively charged MM0.25 surface repels anionic TC species, resulting in reducing adsorption. This dynamic interplay of electrostatic forces accounts for the pH-dependent variation in TC adsorption, with optimal performance achieved near the MM0.25's PZC^45^.

#### Adsorption isotherms

**Fig. 9:**
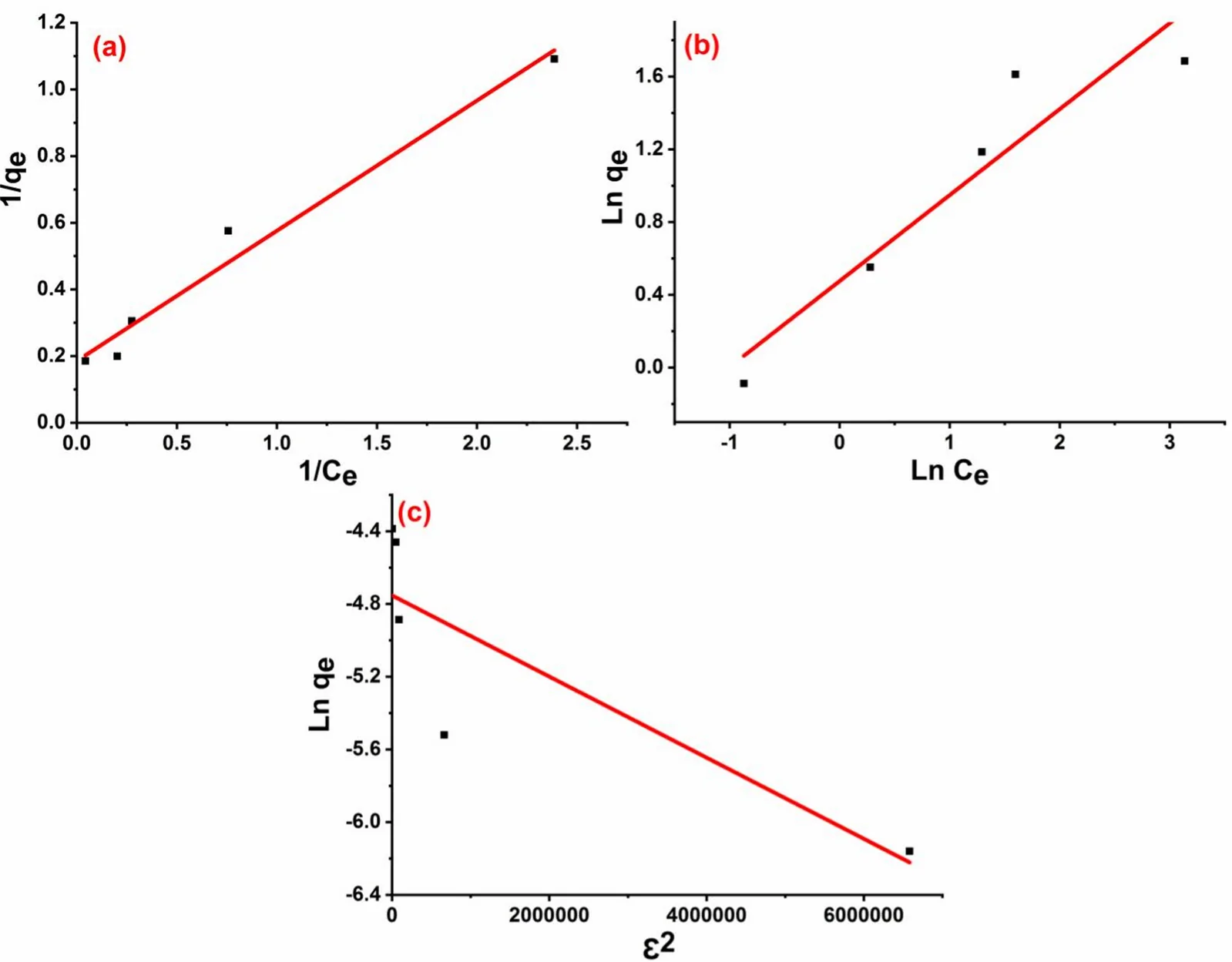
Adsorption isotherms of TC on MM0.25 sample (**a**) Langmuir, (**b**) Freundlich and (**c**) D–R models (5 g L^−1^ adsorbent dose, 10 g L^−1^ TC concentration, pH 5.4 and room temperature).

Figure 9a,b illustrates the fitting results of the Langmuir and Freundlich isotherms alongside the experimental data for TC adsorption on the MM0.25 sample. The corresponding model constants and key correlation coefficients are summarized in Table 2. The suitability of each model was evaluated based on their linear regression (R^2^) values. As indicated by the R^2^ values in Table 2, the adsorption data fit the Langmuir model exceptionally well, signifying the formation of a homogeneous monolayer of TC molecules across both the external and internal surfaces of the adsorbent. Furthermore, this well-fitted behavior implies that competition between the solvent and the adsorbate for active sites was minimal, confirming the high efficiency of the MM0.25 sample for TC removal^46^.

**Table 2:** Adsorption isotherm parameters of Langmuir, Freundlich and D–R isotherm models for TC drug.

Langmuir isotherm model	q_max_ (mg g^−1^)	5.4
	b (L mg^−1^)	0.5
	R^2^	0.97509
Freundlich isotherm model	K_F_ (mg g^−1^) (L mg^−1^)^1/n^	1.6
	n	2.1
	R^2^	0.88714
D–R isotherm model	q_D_ (mmol g^−1^)	115.9
	Β ( mol^2^ kJ^−2^)	2.2E-7
	E (kJ mol^−1^)	1507.6
	R^2^	0.72261

However, there are drawbacks which state that although the synthesized MCM-41 material exhibits a high specific surface area (863 m^2^ g^-1^) and well-ordered mesoporous channels, its maximum adsorption capacity (q_max_ = 5.4 mg g^-1^) is notably low. This performance highlights fundamental surface-chemistry and mass-transport limitations of unfunctionalized silica. First, the pristine silicate matrix is dominated by inert siloxane (Si–O–Si) linkages and isolated silanol (Si–OH) groups. Without post-synthetic functionalization (e.g., amine or carboxyl grafting), the surface lacks high-affinity active binding sites, restricting uptake strictly to weak physisorption^47^. Second, a high surface area does not translate to high capacity when thermodynamic affinity is low; hence, the majority of the internal pore volume remains energetically unfavorable for solute accumulation. Finally, steric constraints and solvent hydration shell barriers within the narrow 3.5 nm mesopores restrict pollutant diffusion into the inner channels, leaving interior active sites underutilized^48^. Comparative studies of the TC adsorption performance of MM0.25 with state-of-the-art MCM-41 and other mesoporous silica adsorbents have been integrated and collected in Table 3.

**Table 3:** Adsorption capacities of some adsorbents compared to MM0.25 for the removal of TC drug.

Adserbent	Dosage (g L^−1^)	pH	t (min)	q_m_ (mg g^−1^)	References
Cu-immobilized alginate	0.05	3	1680	53.3	^50^
Pumice stone	10	3	120	20	^51^
Granular sludge		7.5–8.0	2160	15.2	^52^
Biochar OH	5	2	1440	11.9	^53^
Biochar M	5	2	1440	15.2	^53^
Fe_3_O_4_ magnetized starch polyurethane	2.5	6	240	16.4	^54^
SBA	1	5	1440	41.1	^55^
illite (IMt-2)	10	5–6	1440	32	^56^
Kaolinite				4.3	^57^
Present study	5	5.4	5	5.4	

Dubinin–Radushkevich (D–R) model was used to examine the adsorption process and differentiate between chemical and physical adsorption, according to the following equation^49^:

$$\mathrm{ln}{q}_{e}=ln{q}_{D}-\beta {\varepsilon }^{2}$$

q_e_ is the adsorption capacity of the TC drug on MM0.25 adsorbent at equilibrium (mmol g^-1^); q_D_ is the D–R adsorption capacity (mmol g^-1^); *β * (mol^2^ kJ^-2^) is the activity coefficient depending on average energy of adsorption; *\varepsilon * is the Polanyi potential obtained by the equation:

$$\upvarepsilon =\mathrm{R}\mathrm{T} (1+ \frac{1}{{\mathrm{C}}_{\mathrm{e}}})$$

R is the gas constant (kJ mol^-1^ K^-1^), C_e_ is the equilibrium concentration of TC drug (mol L^-1^) and T is the absolute temperature (K). A straight line is obtained by plotting ln q_e_ vs ε^2^; the slope of this line is *β * and the intercept equal ln q_D_ (Fig. 9c). The average free energy (kJ mol^-1^) could be obtained according to the relation:

$$\mathrm{E}= \frac{1}{\sqrt{2\upbeta }}$$

The value of E (kJ mol^-1^) provides a good sign for the type of adsorption process, whether it is physical or chemical. The calculated average free energy for adsorption of TC on MM0.25 was found to be greater than 8 kJ mol^-1^ (1507.6 kJ mol^-1^) (Table 2) referring to that the adsorption of TC on MM0.25 adsorbent is chemical adsorption^11^.

#### Effect of contact time and adsorption kinetics

**Fig. 10:**
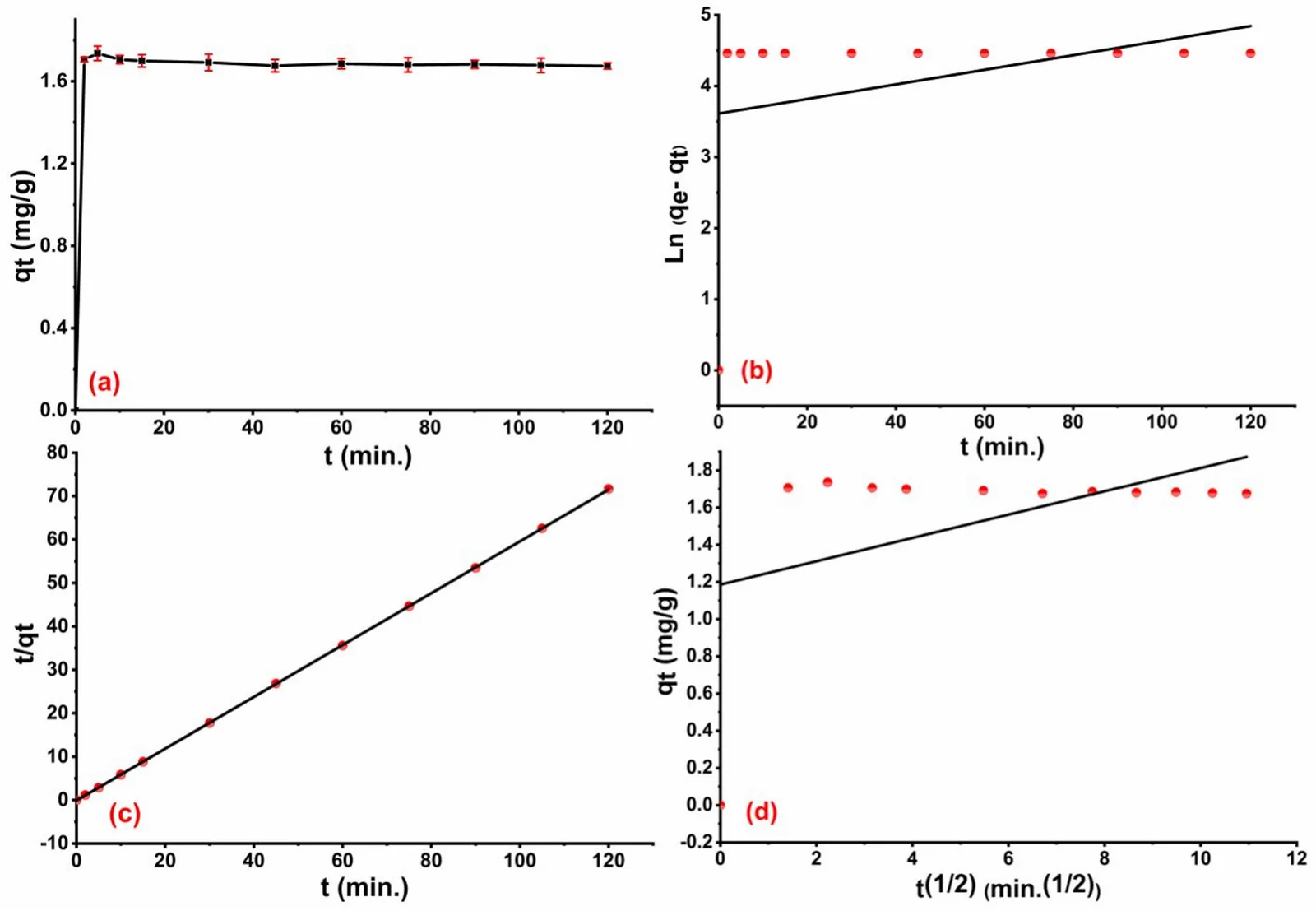
(**a**) Effect of time and (**b**–**d**) kinetic models for adsorption of TC (**b**) pseudo-first order; (**c**) pseudo-second order and (**d**) intraparticle-diffusion (5 g L^−1^ adsorbent dose, 10 g L^−1^ TC concentration, pH 5.4 and room temperature).

As illustrated in Fig. 10a, approximately 86.8% of TC drug was rapidly eliminated by MM0.25 within the first 5 min, after which the adsorption reached equilibrium, as indicated by the plateau in the adsorption curve. This rapid initial adsorption rate can be attributed to the abundance of available active sites on the MM0.25 surface, which established a steep concentration gradient between adsorbed TC molecules on the MM0.25 surface and in the solution. To further evaluate the adsorption kinetics, experiments were conducted at 25 °C with an initial TC concentration of 10 mg L⁻^1^. The experimental data were fitted to pseudo-first-order, pseudo-second-order, and intra-particle diffusion (IPD) models (Fig. 10b–d) to determine the rate parameters and elucidate the underlying reaction mechanism. The calculated kinetic constants and correlation coefficients are summarized in Table 4. Among the models, the pseudo-second-order model provided the best fit (R^2^ = 0.99999), outperforming both the pseudo-first-order and IPD models. This suggests that the adsorption process is predominantly governed by chemisorption, involving the sharing or exchange of electrons between the TC molecules and the MM0.25 surface.

**Table 4:** Kinetic parameters for TC adsorption on MM0.25 sample.

Pseudo-second order	q_2_ (g.mg^−1^.min^−1^)	1.7
	K_2_ (g.mg^−1^_._min^1^)	− 3.98
	R^2^	0.99999
Pseudo-first order	q_1_(mg.g^−1^)	37
	K_1_ (min^−1^)	0.01
	R^2^	0.11658
Intra-particle diffusion	C (mg.g^−1^)	1.2
	K_ipd_ (mg.g^−1^.min^−0.5^)	0.06
	R^2^	0.22303

#### Role of surface properties and interfacial phenomena on adsorption mechanisms

The textural properties and specific surface area play a crucial role in governing the adsorption behavior of mesoporous MCM-41. As evidenced by the N_2_ adsorption–desorption isotherms, MM0.25 sample features an exceptionally high specific surface area (863 m^2^ g^-1^) alongside a well-ordered, uniform mesoporous structure. This extensive surface area provides an abundance of accessible active binding sites and minimizes intraparticle diffusion resistance, thereby facilitating the rapid mass transfer of TC molecules from the aqueous phase to the inner pore surface. Consequently, the high surface area directly contributes to the swift initial adsorption kinetics, achieving 91.6% removal within just 5 min at 5 mg L^-1^, pH 5.4 and room temperature^58,59^.

The adsorption of TC onto the MM0.25 sample is governed by complex interfacial phenomena occurring at the solid–liquid interface^60^. This adsorption mechanism operates through three main synergistic processes: i) attractive electrostatic forces determined by the solution pH relative to the adsorbent's point of zero charge (pH_pzc_) and the dissociation constants (pK_a_) of TC; ii) strong surface hydrogen-bond networks develop between the surface silanol (Si–OH) groups of the silica framework and the polar functional groups (dimethylamino, phenolic hydroxyl, and amide) of the TC molecule; and iii) multi-stage mass transport involving film diffusion across the liquid boundary layer followed by intraparticle diffusion within ordered mesopores^60^. Crucially, the synergy between the large specific surface area and abundant active binding sites minimizes the mass-transfer resistance, resulting in rapid interfacial adsorption kinetics during the initial contact phase.

#### Recovery and regeneration of MM0.25 adsorbent

**Fig. 11:**
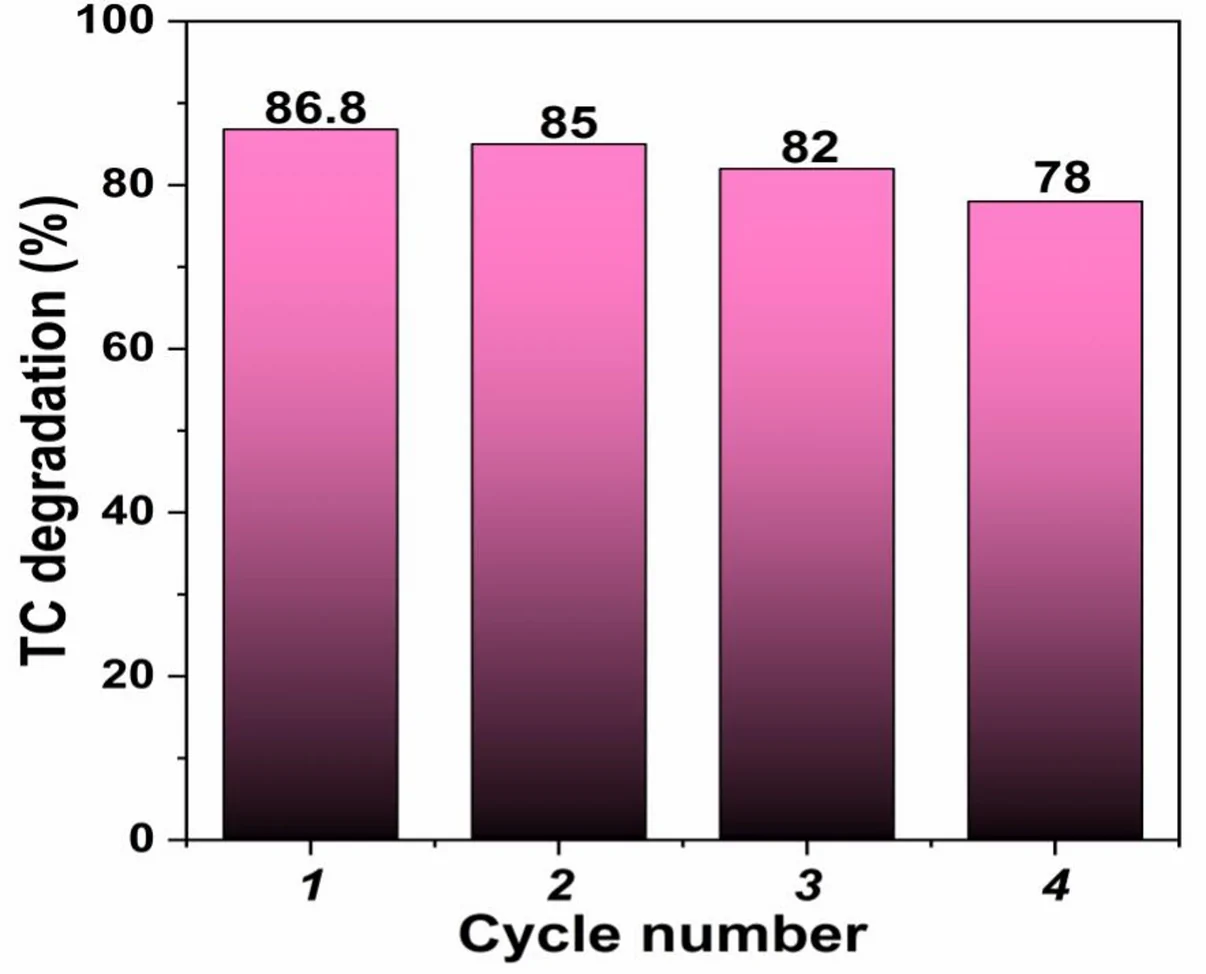
Reusability of MM0.25 for TC removal for four cycles (5 g L^−1^ adsorbent dose, 10 g L^−1^ TC concentration, pH 5.4 and room temperature).

A regeneration study is essential to evaluate the reusability and economic viability of an adsorbent for practical applications. To examine the recovery potential of the MM0.25 adsorbent, recycle experiments were implemented for four subsequent adsorption–desorption cycles. After each experiment, MM0.25 sample was recovered through calcination at 500 °C for 1 h. Then, the recovered sample was reutilized in the next cycle using the same conditions; the TC adsorption results are illustrated in Fig. 11. It could be observed that MM0.25 could adsorb TC efficiently even after reusing for 4 times. The removal percentage somewhat diminished from 86.8% in the first cycle to 78% in the fourth cycle, perhaps because of handling. Overall, the MM0.25 adsorbent could be reused for several cycles while preserving good adsorption activity^11,61,62^.

### Sustainability, environmental impact, material cost-effectiveness and scalability

#### Synthesis sustainability and environmental impact

The synthesis of MM0.25 adsorbent was designed with green chemistry principles, using an aqueous hydrothermal approach to minimize hazardous organic waste generation^63^. Operating primarily in aqueous media reduces the reliance on toxic and volatile organic solvents^64^. Furthermore, the exceptionally high removal rate (91.6% TC removal within 5 min) significantly lower the operational requirements, specifically reducing the required adsorbent dosage, contact time, directly lowering the overall energy during water treatment operations.

#### Material cost-effectiveness

The economic viability is a crucial criterion for scaling up water treatment technologies^65^. Mesoporous silica frameworks such as MCM-41 benefit from abundant, low-cost precursors like sodium silicate or tetraethyl orthosilicate (TEOS). Additionally, the MM0.25 material demonstrates high chemical stability and excellent structural integrity, allowing repeated regeneration without substantial loss in adsorption efficiency. It was observed that MM0.25 could adsorb TC efficiently even after reusing for 4 times (from 86.8% in the first cycle to 78% in the fourth cycle). This strong reusability profile drastically lowers lifetime operating and material replacement costs in practical applications.

#### Scalability

From a practical perspective, evaluating the scalability of autoclave-assisted hydrothermal synthesis is essential for real-world wastewater remediation. Hydrothermal synthesis is already a well-established industrial standard in zeolite and mesoporous silica manufacturing^66,67^. Hydrothermal autoclaves provide precise temperature and pressure control, resulting in high product yield, uniform pore size distribution, and reproducible hexagonal pore geometry^67^.

**Scheme 1:**
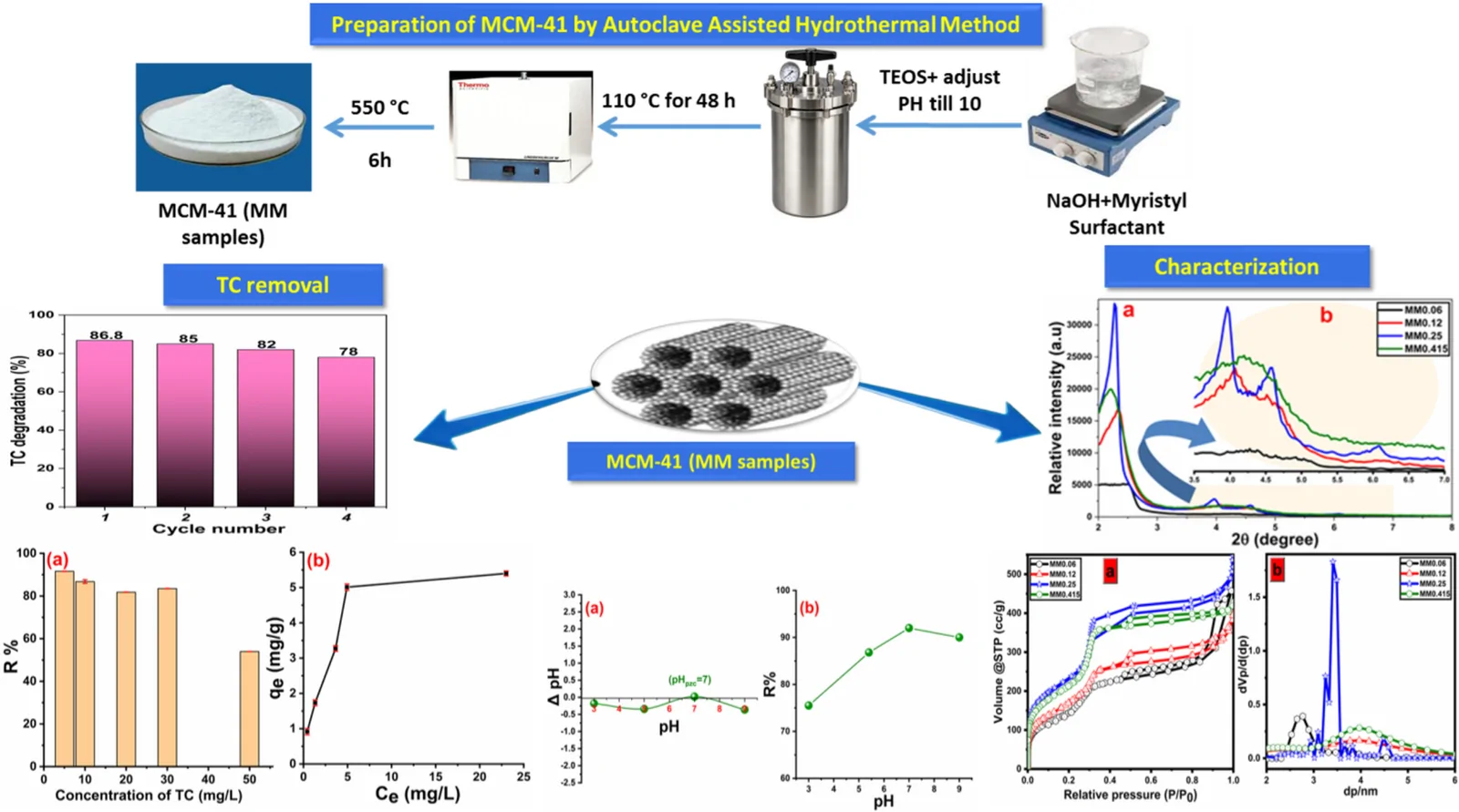
Schematic illustration for preparation, characterization and applications of mesoporous MCM-41 for TC removal.

For real-world wastewater treatment, scaling up MCM-41 synthesis requires optimizing energy efficiency during the crystallization stage (e.g., using microwave-assisted or continuous-flow hydrothermal reactors) and substituting high-purity silica precursors with cost-effective industrial silica sources (e.g., sodium silicate or agricultural ash) (Scheme 1).

## Conclusion

Highly ordered mesoporous MCM-41 was synthesized via an autoclave assisted hydrothermal method. The effect of different molar concentration ratio (0.06, 0.12, 0.25 and 0.415 M) of myristyl trimethyl ammonium bromide surfactant to the silica source was investigated. The synthesized mesoporous MCM-41 were analyzed and characterized using XRD, nitrogen adsorption/desorption, SEM, TEM, and FTIR spectroscopy to establish structure–property relationships by determining the structure, morphology, and surface properties. We can conclude that all analyses confirmed the formation of highly ordered pure MCM-41 for MM0.25 and MM0.12 samples but the MM0.25 sample formed highly ordered pure MCM-41 structure more than the MM0.12 sample. Consequently, the TC adsorption potential of both samples was evaluated. The MM0.25 sample demonstrated rapid adsorption capabilities, achieving a maximum adsorption capacity (q_max_) of 5.4 mg g^-1^ for TC at pH 5.4 and room temperature. Equilibrium data for TC adsorption onto MM0.25 fitted the Langmuir isotherm model well, and the adsorption kinetics followed a pseudo-second-order model (R^2^ = 0.99999). The calculated free adsorption energy obtained from D–R isotherm was found to be 1507.6 kJ mol^-1^ referring to that the adsorption of TC on MM0.25 adsorbent is chemical. These results demonstrate that the MM0.25 sample serves as a highly effective adsorbent for removing TC from aqueous solutions with good recovery and recyclability.

## Supplementary Information

Below is the link to the electronic supplementary material.

## Data Availability

Data availability The datasets used and/ or analysed during the current study available from the corresponding author on reasonable request.

## References

[1] Organization, W.H. *Progress on Household Drinking Water, Sanitation and Hygiene 2000-2022: Special Focus on Gender* (World Health Organization, 2024).

[2] Deb, A., Das, S. & Debnath, A. Fabrication and characterization of organometallic nanocomposite for efficient abatement of dye laden wastewater: CCD optimization, adsorption mechanism, co-existing ions, and cost analysis. *Chem. Phys. Lett.* **830**, 140820 (2023).

[3] Wahba, M. A. et al. V/Zn Incorporated-MCM-41: Synthesis, characterization and visible light photocatalytic activity for Tetracycline degradation. *J. Inorg. Organomet. Polym. Mater* **35**(6), 4445–4464 (2025).

[4] Li, X. et al. Rapid and efficient adsorption of tetracycline from aqueous solution in a wide pH range by using iron and aminoacetic acid sequentially modified hierarchical porous biochar. *Bioreour. Technol.* **346**, 126672 (2022). 10.1016/j.biortech.2022.126672 34998926

[5] Ali, N. S. et al. Studying the kinetics and removal mechanism of the methylene blue dye in a continuous adsorption process using prepared mesoporous materials. *Water Pract. Technol.* **19**(7), 2799–2815 (2024).

[6] Al-Bayati, T. M. J. P. S. Removal of aniline and nitro-substituted aniline from wastewater by particulate nanoporous MCM-48. *Technology* **32**(6), 616–623 (2014).

[7] Neisan, R. S. et al. Adsorption of copper from water using TiO_2_-modified activated carbon derived from orange peels and date seeds: Response surface methodology optimization. *Heliyon* **9**(11), e21420 (2023). 38027893 10.1016/j.heliyon.2023.e21420 PMC10660060

[8] Yang, W. et al. Bio-originated mesosilicate SBA-15: Synthesis, characterization, and application for heavy metal removal. *NPJ Clean Water* **7**(1), 49 (2024).

[9] Beck, J. S. et al. A new family of mesoporous molecular sieves prepared with liquid crystal templates. *J. Am. Chem. Soc.* **114**(27), 10834–10843 (1992).

[10] Ying, J. Y., Mehnert, C. P. & Wong, M. S. Synthesis and applications of supramolecular-templated mesoporous materials. *Angew. Chem. Int. Edn.* **38**(1–2), 56–77 (1999).

[11] Khaled, R. K. et al. Highly ordered pure and indium-incorporated MCM-41 mesoporous adsorbents: Synthesis, characterization and evaluation for dye removal. *J. Mater. Sci.* **57**(7), 4504–4527 (2022).

[12] Yang, G. et al. A facile approach to synthesize MCM-41 mesoporous materials from iron ore tailing: Influence of the synthesis conditions on the structural properties. *Appl. Clay Sci.* **111**, 61–66 (2015).

[13] Corma, A. From microporous to mesoporous molecular sieve materials and their use in catalysis. *Chem. Rev.* **97**(6), 2373–2420 (1997). 11848903 10.1021/cr960406n

[14] Lelong, G. et al. Effect of surfactant concentration on the morphology and texture of MCM-41 materials. *J. Phys. Chem. C* **112**(29), 10674–10680 (2008).

[15] Liu, S. et al. The influence of the alcohol concentration on the structural ordering of mesoporous silica: Cosurfactant versus cosolvent. *J. Phys. Chem. B* **107**(38), 10405–10411 (2003).

[16] Soler-Illia, G. et al. Chemical strategies to design textured materials: from microporous and mesoporous oxides to nanonetworks and hierarchical structures. *Chem. Rev.* **102**(11), 4093–4138 (2002). 12428985 10.1021/cr0200062

[17] Huo, Q. et al. Mesostructure design with gemini surfactants: Supercage formation in a three-dimensional hexagonal array. *Science* **268**(5215), 1324–1327 (1995). 17778977 10.1126/science.268.5215.1324

[18] Rodriguez-Abreu, C. et al. Structural evolution during the synthesis of mesoporous silica in fatty acid/aminoalkoxysilane/water systems. *J. Phys. Chem. B* **108**(52), 20083–20089 (2004).

[19] Zhao, D. et al. Nonionic triblock and star diblock copolymer and oligomeric surfactant syntheses of highly ordered, hydrothermally stable, mesoporous silica structures. *J. Am. Chem. Soc.* **120**(24), 6024–6036 (1998).

[20] Fan, J. et al. Cubic mesoporous silica with large controllable entrance sizes and advanced adsorption properties. *Angew. Chem. Int. Edn.* **42**(27), 3146–3150 (2003). 10.1002/anie.200351027 12866103

[21] Yan, X. et al. Synthesis of highly ordered mesoporous silica using cationic trimeric surfactant as structure-directing agent. *Colloids Surf. A Physicochem. Eng. Asp.* **303**(3), 219–225 (2007).

[22] Narayan, R. et al. Mesoporous silica nanoparticles: A comprehensive review on synthesis and recent advances. *Pharmaceutics* **10**(3), 118 (2018). 30082647 10.3390/pharmaceutics10030118 PMC6160987

[23] Wahba, M. A., Khaled, R. K. & Dawy, M. J. Tailored bimetallic Zn/Ni and Zn/Ag MCM-41 photocatalysts for enhanced visible-light photocatalytic tetracycline degradation. *Sci. Rep.* **15**(1), 5725 (2025). 39962146 10.1038/s41598-025-89522-y PMC11833085

[24] Mansour, F., Dimeo, R. & Peemoeller, H. High-resolution inelastic neutron scattering from water in mesoporous silica. *Phys. Rev. E* **66**(4), 041307 (2002). 10.1103/PhysRevE.66.041307 12443199

[25] Meléndez-Ortiz, H. I. et al. Hydrothermal synthesis of mesoporous silica MCM-41 using commercial sodium silicate. *J. Mexican Chem. Soc.* **57**(2), 73–79 (2013).

[26] Palmqvist, A. E. Synthesis of ordered mesoporous materials using surfactant liquid crystals or micellar solutions. *Curr. Opin. Colloid Interface Sci.* **8**(2), 145–155 (2003).

[27] Kruk, M. et al. Determination of pore size and pore wall structure of MCM-41 by using nitrogen adsorption, transmission electron microscopy, and X-ray diffraction. *J. Phys. Chem. B* **104**(2), 292–301 (2000).

[28] Kruk, M., Jaroniec, M. & Sayari, A. Adsorption study of surface and structural properties of MCM-41 materials of different pore sizes. *J. Phys. Chem. B* **101**(4), 583–589 (1997).

[29] Suvanto, S. et al. High-cobalt-loaded MCM-41 via the gas-phase method. *Langmuir* **16**(9), 4109–4115 (2000).

[30] Wahba, M. A. et al. Enhanced optical and antimicrobial activities of mono Zn and bimetallic (Zn, Co),(Zn, Pd) ions modified MCM-41: Structural and morphological investigation. *J. Porous Mater.* **31**(5), 1915–1931 (2024).

[31] Wu, Y. et al. Pore size control of monodisperse mesoporous silica particles with alkyl imidazole ionic liquid templates for high performance liquid chromatography applications. *Colloids Surf. A Physicochem. Eng. Asp.* **637**, 128200 (2022).

[32] Zhou, Y. Y., Li, X. X. & Chen, Z. X. Rapid synthesis of well-ordered mesoporous silica from sodium silicate. *Powder Technol.* **226**, 239–245 (2012).

[33] Vazquez, N. I. et al. Synthesis of mesoporous silica nanoparticles by sol–gel as nanocontainer for future drug delivery applications. *Boletín de la Sociedad Española de Cerámica y Vidrio* **56**(3), 139–145 (2017).

[34] Purwaningsih, H. et al. Effect of cetyl trimethyl ammonium bromide as template of mesoporous silica mcm-41 from rice husk by sol-gel method. In *IOP Conference Series: Materials Science and Engineering* (IOP Publishing, 2019).

[35] Zhao, D., Wan, Y. & Zhou, W. *Ordered Mesoporous Materials* (John Wiley & Sons, 2012).

[36] Li, K. et al. Preparation of Physical-modified Mesoporous Silica. In *IOP Conference Series: Earth and Environmental Science* (IOP Publishing, 2021).

[37] Purwaningsih, E. Struktur kepemilikan memoderasi pengaruh profitabilitas terhadap kebijakan dividen. *Jurnal Ekonomi Universitas Esa Unggul* **10**(2), 111–120 (2019).

[38] Tunç, S. et al. Rheological measurements of Na-bentonite and sepiolite particles in the presence of tetradecyltrimethylammonium bromide, sodium tetradecyl sulfonate and Brij 30 surfactants. *Colloids Surf. A Physicochem. Eng. Asp.* **398**, 37–47 (2012).

[39] Khalil, K. M. Cerium modified MCM-41 nanocomposite materials via a nonhydrothermal direct method at room temperature. *J. Colloid Interface Sci.* **315**(2), 562–568 (2007). 17714729 10.1016/j.jcis.2007.07.030

[40] Yun, G.-N. et al. Infrared spectroscopic studies of the hydrodeoxygenation of γ-valerolactone on Ni2P/MCM-41. *Catal. Today* **323**, 54–61 (2019).

[41] Abbasi, K. K. et al. Enhanced gas permeation performance of mixed matrix membranes containing polysulfone and modified mesoporous MCM-41. *J. Serb. Chem. Soc.* **86**(9), 871–887 (2021).

[42] Aksu, Z. & Kabasakal, E. Batch adsorption of 2, 4-dichlorophenoxy-acetic acid (2, 4-D) from aqueous solution by granular activated carbon. *Sep. Purif. Technol.* **35**(3), 223–240 (2004).

[43] Shao, Y. et al. Application of Mn/MCM-41 as an adsorbent to remove methyl blue from aqueous solution. *J. Colloid Interface Sci.* **429**, 25–33 (2014). 24935186 10.1016/j.jcis.2014.05.004

[44] Gao, G. L. et al. Facile construction of magnetic M-ZnO-Ni-RSA composite as a functional photocatalyst for tetracycline degradation: performance and mechanism. *J. Photochem. Photobiol. A Chem.* **448**, 115249 (2024).

[45] Wahba, M., Khaled, R. & Dawy, M. Tailored bimetallic Zn/Ni and Zn/Ag MCM-41 photocatalysts for enhanced visible-light photocatalytic tetracycline degradation. *Sci. Rep.* **15**, 5725 (2025). 39962146 10.1038/s41598-025-89522-y PMC11833085

[46] Huang, X. et al. An innovative approach to recycle boron waste by mesoporous silica production and its application in methylene blue removal. *J. Environ. Chem. Eng.* **9**(4), 105327 (2021).

[47] Williams, J. J. et al. Effect of surface group functionalization on the CO2/N2 separation properties of MCM-41: A grand-canonical Monte Carlo simulation study. *J. Phys. Chem. C* **114**(43), 18538–18547 (2010).

[48] Ajumobi, O. et al. Design of nanostraws in amine-functionalized MCM-41 for improved adsorption capacity in carbon capture. *Energy Fuels* **37**(16), 12079 (2023). 37609064 10.1021/acs.energyfuels.3c01318 PMC10441579

[49] Dao Sy Duc, D. S., Nguyen Bin, N. B. & Tran Thi Huyen Trang, T. T. Adsorption of basic yellow 28 in aqueous solution by activated carbon. *Asian J. Chem.* **25**, 2173 (2013).

[50] Zhang, X. et al. Study on adsorption of tetracycline by Cu-immobilized alginate adsorbent from water environment. *Int. J. Biol. Macromol.* **124**, 418–428 (2019). 30496862 10.1016/j.ijbiomac.2018.11.218

[51] Guler, U. A. & Sarioglu, M. Removal of tetracycline from wastewater using pumice stone: Equilibrium, kinetic and thermodynamic studies. *J. Environ. Health Sci. Eng.* **12**(1), 79 (2014). 24936305 10.1186/2052-336X-12-79 PMC4038404

[52] Shi, Y.-J. et al. Sorption and biodegradation of tetracycline by nitrifying granules and the toxicity of tetracycline on granules. *J. Hazard. Mater.* **191**(1–3), 103–109 (2011). 21570181 10.1016/j.jhazmat.2011.04.048

[53] Antón-Herrero, R. et al. Comparative adsorption of tetracyclines on biochars and stevensite: Looking for the most effective adsorbent. *Appl. Clay Sci.* **160**, 162–172 (2018).

[54] Okoli, C. P. & Ofomaja, A. E. Development of sustainable magnetic polyurethane polymer nanocomposite for abatement of tetracycline antibiotics aqueous pollution: Response surface methodology and adsorption dynamics. *J. Clean. Prod.* **217**, 42–55 (2019).

[55] Zhang, Z., Li, H. & Liu, H. Insight into the adsorption of tetracycline onto amino and amino–Fe3+ gunctionalized mesoporous silica: Effect of functionalized groups. *J. Environ. Sci.* **65**, 171–178 (2018). 10.1016/j.jes.2016.10.020 29548388

[56] Chang, P.-H. et al. Adsorption of tetracycline on 2: 1 layered non-swelling clay mineral illite. *Appl. Clay Sci.* **67**, 158–163 (2012).

[57] Li, Z. et al. Adsorption of tetracycline on kaolinite with pH-dependent surface charges. *J. Colloid Interface Sci.* **351**(1), 254–260 (2010). 20708739 10.1016/j.jcis.2010.07.034

[58] Chen, J. et al. Rapid antibiotic adsorption from water using MCM-41-based material. *Water* **15**(22), 4027 (2023).

[59] Macedo, V. M. et al. Insights on the synthesis of Al-MCM-41 with optimized Si/Al ratio for high-performance antibiotic adsorption. *ACS Omega* **8**(50), 48181 (2023). 38144102 10.1021/acsomega.3c07119 PMC10733947

[60] Mahdi, A. et al. Investigation of equilibrium, isotherm, and mechanism for the efficient removal of 3-nitroaniline dye from wastewater using mesoporous material MCM-48. *Prog. Color Colorants Coat.* **16**(4), 387–398 (2023).

[61] Shirazian, S. et al. Eco-friendly synthesis of crown-ether functionalized silica nanospheres from sorghum waste for thallium adsorption. *J. Taiwan Inst. Chem. Eng.* **167**, 105851 (2025).

[62] Alsaab, H. O. et al. Sustainable synthesis and dual adsorption of methyl orange and cadmium ions using biogenic silica-based fibrous silica functionalized with crown ether ionic liquid. *J. Colloid Interface Sci.* **679**, 555–568 (2025). 39471584 10.1016/j.jcis.2024.10.103

[63] Anastas, P. & Eghbali, N. Green chemistry: Principles and practice. *Chem. Soc. Rev.* **39**(1), 301–312 (2009). 20023854 10.1039/b918763b

[64] Ndlwana, L. et al. Sustainable hydrothermal and solvothermal synthesis of advanced carbon materials in multidimensional applications: a review. *Materials* **14**(17), 5094 (2021). 34501183 10.3390/ma14175094 PMC8434334

[65] Mileta, S. & Nuić, I. J. S. Towards sustainable water treatment: From adsorption to regeneration and end-of-life management of heavy metal-loaded biosorbents. *Sustainability.* **18**(13), 6673 (2026).

[66] Brankovic, M. et al. Mesoporous silica (MCM-41): Synthesis/modification, characterization and removal of selected organic micro-pollutants from water. *Adv Technol.* **6**(1), 50–57 (2017).

[67] Kresge, C. T. & Roth, W. J. The discovery of mesoporous molecular sieves from the twenty year perspective. *Chem. Soc. Rev.* **42**(9), 3663–3670 (2013). 23426610 10.1039/c3cs60016e

