# Supplementary material for: Tailoring the structural and textural properties of MCM-41 via myristyl trimethyl ammonium bromide templating for enhanced tetracycline adsorption
Source: Sci Rep. 2026 Sep 2;16:27558. doi: 10.1038/s41598-026-67402-3 (PMC13538651; doi:10.1038/s41598-026-67402-3)
Supplement: Supplementary file 1 [file 41598_2026_67402_MOESM1_ESM.docx]

Figure S1: The calibration curve of TC drug.
